# A method for extracting high-quality total RNA from plant rich in polysaccharides and polyphenols using *Dendrobium huoshanense*

**DOI:** 10.1371/journal.pone.0196592

**Published:** 2018-05-01

**Authors:** Lulu Liu, Rongchun Han, Nianjun Yu, Wei Zhang, Lihua Xing, Dongmei Xie, Daiyin Peng

**Affiliations:** 1 School of Pharmacy, Anhui University of Chinese Medicine, Hefei, China; 2 Anhui Innovative Team from Colleges for Scientific Research's Platform—The Innovative Team in Researching the Key Technologies Concerning the Integration of Processing Chinese Medicine Decoction Pieces in Producing Area, Hefei, China; University of Western Sydney, AUSTRALIA

## Abstract

Acquiring high quality RNA is the basis of plant molecular biology research, plant genetics and other physiological investigations. At present, a large number of nucleotide isolation methods have been exploited or modified, such as commercial kits, CTAB, SDS methods and so on. Due to the nature of different plants, extraction methods vary. Moreover, efficiency of certain approach cannot be guaranteed due to composition of different plants and extracting high quality RNA from plants rich in polysaccharides and polyphenols are often difficult. The physical and chemical properties of polysaccharides which are similar to nucleic acids and other secondary metabolites will be coprecipitated with RNA irreversibly. Therefore, how to remove polysaccharides and other secondary metabolites during RNA extraction is the primary challenge. *Dendrobium huoshanense* is an Orchidaceae perennial herb that is rich in polysaccharides and other secondary metabolites. By using *D*. *huoshanense* as the subject, we improved the method originated from CHAN and made it suitable for plants containing high amount of polysaccharides and polyphenols. The extracted total RNA was clear and non-dispersive, with good integrity and no obvious contamination with DNA and other impurities. And it was also evaluated by gel electrophoresis, nucleic acid quantitative detector and PCR assessment. Thus, as a simple approach, it is suitable and efficient in RNA isolation for plants rich in polysaccharides and polyphenols.

## Introduction

*Dendrobium huoshanense* C. Z. Tang et S. J. Cheng is a famous Orchidaceae perennial medicinal herb that has been used to remedy extensive symptoms, such as chronic superficial gastritis, throat phlegmonosis, to enhance immunity and so forth [1]. *D*. *huoshanense* is a precious medicinal herb in China, and rare Daodi herb [2]. Because of its low yield and excellent efficacy, high demand from the market resulted in this endangered species [3]. The government made efforts to protect and develop this herbal medicine, including providing low-interest loans, increasing investment in greenhouse cultivation, and as a result, studies on breeding, large-scale planting and imitation of wild cultivation are on the way [2,4–7]. *D*. *huoshane*nse contains a variety of chemical components such as polysaccharides [8], alkaloids [9], amino acids [10], flavonoids [11] and so on. Modern pharmacological studies have shown that *D*. *huoshanense* have the efficacy of enhancing immunity [12], anti-oxidation [13], anti-tumor [10], cell apoptosis inhibition [14], lowering blood glucose [15], liver protection, [16] etc. At present, the research on *D*. *huoshanense* mainly focuses on germplasm resources, cultivation techniques and pharmacological effects.

Obtaining high quality RNA is the basis for many plant molecular biology researches [17]. RT-PCR, cDNA synthesis and subsequent genetic analysis all require RNA with high purity and integrity. A particularly thorny problem regarding RNA extraction of *D*. *huoshanense* is its high content of polysaccharides [18–20]. Studies found that in its stem water-soluble polysaccharides were accounted for more than 90% of the total polysaccharide [21] and total polysaccharides in *D*. *huoshanense* stem took up 36.23 per cent of its dry weight [22]. However, due to similarity in physical and chemical properties between RNA and polysaccharides, it can coprecipitate with RNA during nucleotide purification process [23]. Moreover, polysaccharides, polyphenols and other secondary metabolites interfere with or degrade RNA [17]. Therefore, removing polysaccharide effectively is the key to extract high quality RNA from *D*. *huoshanense* and other plants with similar traits. In this study, *D*. *huoshanense* was selected as the subject to extract total RNA from its stem, leaf and flower by applying modified CHAN method [24–25]. In addition, Trizol (Lot no. 135404, Invitrogen, USA), RNeasy Plant Mini Kit (Lot no. 154048665, Qiagen, Germany) and RNAprep Pure Plant Kit (Lot no. Q5510, Tiangen, China) were also used to provide more data for the comparison regarding efficiency of different approaches. Here, we provide a simple, economical and efficient RNA extraction method for plants rich in polysaccharides and polyphenols.

## Materials and methods

### Plant materials

Subjects were collected from National Breeding Base of Seeds and Seedling of Medical Herbs for Essential Drugs (Anhui). Professor Nianjun Yu from Anhui University of Chinese Medicine identified the samples as *D*. *huoshanense* C. Z. Tang et S. J. Cheng from Orchidaceae. Stems, leaves and flowers of *D*. *huoshanense* were washed with tap water, then sterilized with 75% ethanol for 45s, and then rinsed twice with sterile water. Subsequently, plant organs were placed on a sterile filter paper to remove water and finally ground rapidly in liquid nitrogen to obtain fine powder for RNA extraction.

### Solutions and regents

Extraction buffer (EB) (0.25M, NaCl; 0.05M, Tris-HCl (pH = 7.5); 20mM, EDTA; 1%(w/v) sodium dodecyl sulphate) was modified from the protocol proposed by CHAN [24] and PVP (M.W.40.000) was added to EB to the final concentration of 4%(w/v). In addition, Chloroform: Isoamyl alcohol (CI, 24:1 v/v) and Phenol: Chloroform isoamyl alcohol (PCI, 1:1 v/v) were used [25].

### RNA isolation

1. 75μL EB, 750μL CI and 30μL β-mercaptoethanol were mixed in a 2.0mL RNase-free microcentrifuge tube.
2. 50~100 mg of tissue powder made by grinding *D*. *huoshanense* stem, leave and flower respectively in liquid nitrogen were added to tubes. Vortex vigorously. Place at 20°C for 5 minutes before centrifuging the samples at 4°C for 5 min at 12000×g. Note that the samples should not thaw when they were added to solutions prepared in step 1.
3. The supernatant (~700μL) was transferred to a new 2.0mL RNase-free microcentrifuge tube and 700μL of PCI was then added. Mix gently and centrifuge at 4°C for 5 min at 12000×g.
4. The supernatant (~600μL) was transferred to a new 2.0mL RNase-free microcentrifuge tube. 600μL of PCI was added. Mix and centrifuge at 4°C for 5 min at 12000×g. This step resulted in ca 400 μL of supernatant.
5. 1/10 volume of 3M sodium acetate (pH = 5.2) and 2.5 volume of absolute ethanol were added to the supernatant. Mix gently and incubate at 4°C for 30 minutes.
6. Centrifuge at 4°C for 10 min at 12000×g to recover the nucleic acids.
7. 200 μL of DEPC-treated water was added to dissolve the nucleic acids and 500μL of 10M LiCl was added to the solution. Mix gently and then place on ice for 60 minutes.
8. Centrifuge at 4°C for 10 min at 12000×g. Discard the supernatant and wash the pellet with 800μL of 75% (V/V) ethanol. Air-dry in a ventilator for 5-10min.
9. 50 μL of DEPC-treated water was added to dissolve RNA. Incubate at 50°C for 5 minutes. RNA samples can be used immediately for downstream experiments or stored at -80°C for future use.

### RNA analysis

The total RNA extract of stem, leaf and flower of *D*. *huoshanense* were collected and tested for integrity by 1.0% (w/v) agarose gel. A_260/230_ and A_260/280_ values for RNA samples were measured with an Analytic JENA Scandrop 200 to evaluate purity and yield.

### First-strant cDNA synthesis and PCR amplification

The first strand cDNA was synthesized using FastQuant RT kit (TIANGEN BIOTECH, BEIJING) according to the manufacturer’s instructions. Through BLAST alignment, tubulin homologous sequences were searched in our *D*. *huoshanensis* transcriptomic data. Primer 5.0 software was used to design primers for specific PCR amplification. Sangon Biotech synthesized the sequence. The forward primer of tubulin was 5’-TTC TGG GAG GTG ATC TGC GA-3’ and the reverse 5’-CGG GGG AAT GGA ATG AGG TT-3’. PCR reaction system: 33.5μL ddH2O, 5μL 10× Extaq buffer, 5μL 2.5mM dNTP Mix, 2μL cDNA, 2μL 10μM Forward Primer, 2μL 10μM Reverse Primer and 0.5μL Extaq enzyme. PCR reaction conditions: initial denaturation at 94°C, 2min; 30 cycles at 94°C for 30s, 52°C for 30s and 72°C for 2min. Later, 10μL of the PCR product was used for agarose electrophoresis using TBE buffer (1×) following the usual procedure. The remaining products were stored at -20°C.

## Results and discussion

Plant gene expression analysis, cloning, transcriptomic and other research often need to isolate and purify RNA from tissues and high purity, high concentration and good integrity RNA is always a must [17, 26]. At present, a large number of methods have been exploited or modified [27–30]. But plants have large differences in composition and this requires suitable approaches for different types of plants. Moreover, the efficiency of certain method to extract RNA from distinct plants is not the same. As the common knowledge, extracting high quality RNA from plants rich in polysaccharides and polyphenols is very difficult. The physical and chemical properties of polysaccharides are similar to those of RNA [23], so co-precipitation of polyphenols easily occurs and thus RNA quality is inevitably compromised [31]. Studies have shown that *D*. *huoshanense* contains many polysaccharides, phenols, proteins and other secondary metabolites [8–11]. These substances not only reduce RNA yield but also degrade RNA. Conventional RNA extraction methods are difficult to extract high quality RNA from *D*. *huoshanense*. By trying CHAN method [25], Trizol, RNeasy Plant Mini Kit and RNAprep Pure Plant Kit to extract *D*. *huoshanense* RNA, the results were not ideal and the price for purchasing commercial kits was relatively high. Therefore, we set out to establish a simple, economical and efficient RNA extraction method in order to acquire high-quality RNA from plant rich in polysaccharides and polyphenols.

**Fig 1:**
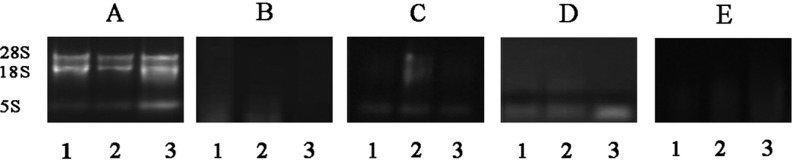
1.0% agarose gel electrophoresis of total RNA isolated. A, three intact RNA bands for 28S, 18S and 5S RNA. Lane 1, lane 2 and lane 3 in A, B, C, D and E contain 1 μg of total RNA from *D*. *huoshanense* stem, leaf and flower, respectively. A: modified CHAN method; B: original CHAN method; C: Trizol method; D: RNeasy Plant Mini Kit method; E: RNAprep Pure Plant Kit method.

In this study, extracted total RNA was verified by gel electrophoresis, quantitative nucleic acid detection and PCR analysis. As a routine examination method [24–32], all samples were tested by 1.0% of modified agarose gel electrophoresis. From Fig 1A, 28 s and 18 s and 5 s stripes were clear with high brightness and no obvious towing degradation phenomenon, which suggested that this modified method from CHAN assured RNA integrity. On the contrary, the bands resulted from original CHAN, Trizol, RNeasy Plant Mini Kit and RNAprep Pure Plant Kit were blurred and the obvious trailing showed phenomenon of degradation (Fig 1B, Fig 1C, Fig 1D and 1E). During extraction process, by applying original CHAN, Trizol, RNeasy Plant Mini Kit and RNAprep Pure Plant Kit methods, the supernatants were dense and viscous, suggesting their poor performance in removing inherent polysaccharides and polyphenols.

The concentration of total RNA was detected. OD value as one of the means of detecting the purity of RNA has been widely used [26–31]. Under neutral pH condition, RNA has maximum absorption at 260nm and according to absorbance at this wavelength RNA concentration in the solution can be determined. The ratio between A_260_ and A_280_ reveals seriousness of protein contamination. Likewise, A_260_/A_230_ provides clues for polysaccharides and other related metabolites contamination. Using the modified CHAN method, results from nucleic acid quantitative analyzer showed A_260_/A_280_ value of stem RNA at around 2.0, A_260_/A_230_ between 1.43 and 2.14 and the concentration ranged from 54.31 to 122.89 ng/μL (Table 1). For total RNA extracted from leaf, A_260_/A_280_ values were at about 2.0, A_260_/A_230_ at around 2.0 and the concentration from 257.52 to 297.31 ng/μL (Table 1). For *D*. *huoshanense* flower, A_260_/A_280_ values at around 2.0, A_260_/A_230_ between 1.99 and 2.06 and the concentration ranged from 261.09 to 585.72 ng/μL (Table 1). The data showed that the modified CHAN method was suitable for extracting high quality total RNA from leaf, stem and flower of *D*. *huoshanense* without serious protein and other impurities pollution. From Table 1, RNA concentration from stem seemed to be lower than that of leaf and flower. This may be because its stem contained more water compared with leaf and flower. From Table 1, Table 2, Fig 1A, and Fig 1B, compared with the original, modified CHAN method performed much better as far as concentration and purity were concerned. The elevated concentration of LiCl may play a key role in removing excess secondary metabolites. From Table 3, RNA concentration by Trizol method was high but OD_260/230_ value suggested severe polysaccharide contamination. Poor quality of total RNA resulted from RNeasy Plant Mini Kit (S1 Table) and RNAprep Pure Plant Kit (S2 Table) method suggested such kits were not suitable to purify RNA from *D*. *huoshanense*. The Kit method performed poorly in obtaining qualified RNA with very low concentration. Thus, the modified CHAN method is more suitable for *D*. *huoshanense* RNA extraction.

**Table 1:** Concentration and purity of total RNA isolated from *D*.*huoshanense* stem, leaf and flower using the modified CHAN method.

No.	Plant organ	A_260/280_	A_260/230_	Concentration(ng/μL)
1	Dh-stem	1.97	1.43	122.89
2	Dh-stem	1.99	2.14	54.31
3	Dh-stem	1.98	1.77	94.20
4	Dh-leaf	2.01	2.19	297.31
5	Dh-leaf	2.05	1.96	257.52
6	Dh-leaf	1.89	2.00	282.20
7	Dh-flower	1.99	1.88	316.29
8	Dh-flower	2.06	2.19	585.72
9	Dh-flower	1.91	2.10	261.09

**Table 2:** Concentration and purity of total RNA isolated from *D*.*huoshanense* stem, leaf and flower using original CHAN method.

No.	Plant organ	A_260/280_	A_260/230_	Concentration(ng/μL)
1	Dh-stem	1.20	1.55	-44.25
2	Dh-stem	-0.10	-0.03	-0.94
3	Dh-stem	0.76	0.11	6.79
4	Dh-leaf	0.77	0.13	8.38
5	Dh-leaf	1.11	0.20	18.12
6	Dh-leaf	-0.15	-0.03	-1.20
7	Dh-flower	0.93	0.87	22.90
8	Dh-flower	1.40	0.19	2.41
9	Dh-flower	-2.34	-0.19	-8.42

**Table 3:** Concentration and purity of total RNA isolated from *D*.*huoshanense* stem, leaf and flower using Trizol method.

No.	Plant organ	A_260/280_	A_260/230_	Concentration(ng/μL)
1	Dh-stem	1.53	0.34	266.22
2	Dh-stem	1.90	0.16	110.85
3	Dh-stem	1.90	0.15	112.58
4	Dh-leaf	1.59	0.37	324.08
5	Dh-leaf	2.38	0.68	5527.80
6	Dh-leaf	2.46	0.68	5231.41
7	Dh-flower	1.53	0.49	95.31
8	Dh-flower	2.02	0.19	152.85
9	Dh-flower	1.98	0.40	87.67

**Fig 2:**
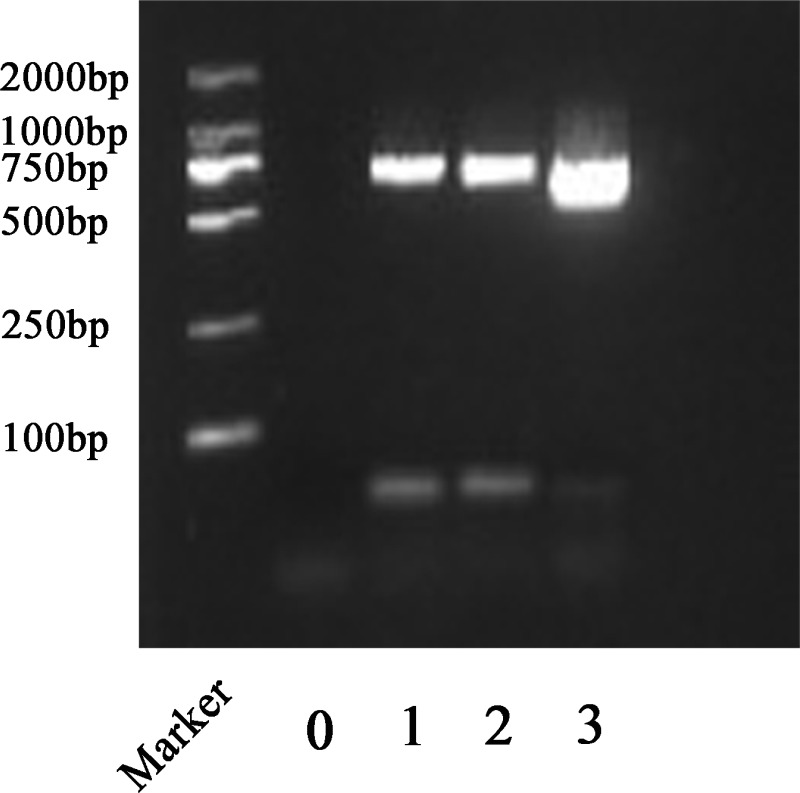
Agarose gel electrophoresis of the RT-PCR tubulin products. Lane 1, lane 2 and lane 3 showed RT-PCR products from *D*. *huoshanense* stem, leaf and flower. Lanes 0 showed RT-PCR product by substituting *D*. *huoshanense* cDNA for ddH_2_O. Marker: DL2,000 DNA marker.

The quality of total RNA was detected by polymerase chain reaction. RNA samples were reverse transcribed into cDNA using FastQuant RT kit and the resultant cDNA was amplified using PCR. From the agarose gel electrophoresis in Fig 2, cDNA of stem, leaf and flower could be amplified by RT-PCR to obtain a target fragment of approximately 720 bp in size, in line with the expected result. This demonstrated that total RNA extracted from *D*. *huoshanense* by the modified CHAN method could meet the requirements of related molecular biological researches.

In general, the process of RNA extraction can be completed in two and a half hours, which is time-saving. During the course of RNA purification, the prevention of RNA degradation should be also taken into consideration. Liquid nitrogen was used to provide low temperature environment [33], inhibit RNA enzymes in low temperature and prevent RNA from degradation in grinding process. Furthermore, in order to avoid degradation by RNase in solutions used for RNA purification, a final concentration of 0.1% DEPC were prepared for all aqueous solutions. Moreover, β-mercaptoethanol was added to the extraction buffer to the final concentration of 2% (v/v) as a strong reducing agent. β-mercaptoethanol is commonly used as an antioxidant in biological experiments, and can inhibit the enzyme activity of RNA [24,28]. The use of SDS prompted nucleoprotein complex dissociation, RNA and protein separation and this would enhance the efficiency of RNA purification. Adding PVP can effectively reduce the interference of phenols on the experiment and PVP in the liquid could also combine with polysaccharides to help remove them [25, 33]. The use of phenol, chloroform and isoamyl-alcohol effectively removed proteins. Moreover, the high concentration of LiCl is effective in removing polysaccharides while precipitating RNA [24, 25, 31]. The modified CHAN method is quick, cheap and especially suitable for plants with high content of polysaccharides.

## Conclusion

By using the modified CHAN method, we also extracted total RNA from leaves of *Salvia miltiorrhiza* and *Platycodon grandiflorus*, resulting in high quality total RNA (Table 4). The essence of this approach lies in the fact that polysaccharides can be efficiently removed, which is very hard for other conventional method to tackle. We succeeded in isolating high quality RNA from *D*. *huoshanense*. And the nucleic acids extracted by this method were verified by gel electrophoresis, quantitative nucleic acid detection and RT-PCR. In conclusion, the method is suitable for extracting RNA from plants rich in polysaccharides.

**Table 4:** Concentration and purity of total RNA isolated from leaves of *Salvia miltiorrhiza* and *Platycodon grandiflorus* using modified CHAN method.

No	Sm-leaf			Pg-leaf		
	A_260/280_	A_260/230_	C(ng/μL)	A_260/280_	A_260/230_	C(ng/μL)
1	1.95	1.77	613.44	2.11	2.46	576.18
2	2.06	1.96	557.13	1.92	1.80	457.64
3	1.87	2.06	478.71	1.99	1.75	151.78

## Supporting information

S1 Table Concentration and purity of total RNA isolated from *D*.*huoshanense* stem, leaf and flower using RNeasy Plant Mini Kit method. (DOCX)

S2 Table Concentration and purity of total RNA isolated from *D*.*huoshanense* stem, leaf and flower using RNAprep Pure Plant Kit method. (DOCX)
