# Supplementary material for: A method for extracting high-quality total RNA from plant rich in polysaccharides and polyphenols using Dendrobium huoshanense
Source: PLoS One. 2018 May 1;13(5):e0196592. doi: 10.1371/journal.pone.0196592 (PMC5929529; doi:10.1371/journal.pone.0196592)
Supplement: S1 Table — (DOCX) [file pone.0196592.s001.docx]

**S1 Table. Concentration and purity of total RNA isolated from *D. huoshanense* stem, leaf and flower using RNeasy Plant Mini Kit method.**

| No. | Plant organ | A_260/280_ | A_260/230_ | Concentration（ng/µL) |
| --- | --- | --- | --- | --- |
| 1 | Dh-stem | 2.07 | 0.73 | 15.48 |
| 2 | Dh-stem | 2.11 | 0.05 | 7.13 |
| 3 | Dh-stem | 1.82 | 0.09 | 3.33 |
| 4 | Dh-leaf | 2.69 | 0.03 | 1.65 |
| 5 | Dh-leaf | 1.93 | 0.30 | 6.10 |
| 6 | Dh-leaf | 3.39 | 0.02 | 5.00 |
| 7 | Dh-flower | 2.49 | 0.04 | 5.43 |
| 8 | Dh-flower | 2.10 | 1.05 | 33.47 |
| 9 | Dh-flower | 1.06 | -0.47 | -1.49 |
