# Supplementary material for: A method for extracting high-quality total RNA from plant rich in polysaccharides and polyphenols using Dendrobium huoshanense
Source: PLoS One. 2018 May 1;13(5):e0196592. doi: 10.1371/journal.pone.0196592 (PMC5929529; doi:10.1371/journal.pone.0196592)
Supplement: S2 Table — (DOCX) [file pone.0196592.s002.docx]

**S2 Table. Concentration and purity of total RNA isolated from *D. huoshanense* stem, leaf and flower using RNAprep Pure Plant Kit method.**

| No. | Plant organ | A_260/280_ | A_260/230_ | Concentration（ng/µL) |
| --- | --- | --- | --- | --- |
| 1 | Dh-stem | 2.10 | 0.14 | 14.22 |
| 2 | Dh-stem | 1.98 | 0.13 | 30.71 |
| 3 | Dh-stem | -2.80 | 0.24 | 15.62 |
| 4 | Dh-leaf | 3.17 | 0.05 | 6.10 |
| 5 | Dh-leaf | 11.12 | 0.39 | 54.64 |
| 6 | Dh-leaf | -5.88 | 0.08 | 5.99 |
| 7 | Dh-flower | 1.57 | 0.31 | 59.46 |
| 8 | Dh-flower | 1.66 | 0.23 | 31.62 |
| 9 | Dh-flower | 2.48 | 0.09 | 20.25 |
